# Organic matter removal in a simultaneous nitrification–denitrification process using fixed-film system

**DOI:** 10.1038/s41598-022-05521-3

**Published:** 2022-02-03

**Authors:** P. González-Tineo, A. Aguilar, A. Reynoso, U. Durán, M. Garzón-Zúñiga, E. Meza-Escalante, L. Álvarez, D. Serrano

**Affiliations:** 1 Departamento de Ciencias del Agua y Medio Ambiente, Instituto Tecnológico de Sonora, Calle 5 de febrero 818 Sur. Col. Centro. Cd. Obregón, Sonora, Mexico; 2 Instituto de Ingeniería, UNAM, P.O. Box 70-186, 04510 México City, Mexico; 3 Instituto Politécnico Nacional (IPN) CIIDIR-DURANGO, Sigma 119, 20 de noviembre II, 34220 Durango, Mexico; 4 Departamento de Ciencias Agronómicas y Veterinarias, Instituto Tecnológico de Sonora, Cuidad Obregón, Mexico

**Keywords:** Environmental impact, Biotechnology, Molecular biology, Environmental sciences

## Abstract

Swine wastewater treatment is a complex challenge, due to the high organic matter (OM) and nitrogen (N) concentrations which require an efficient process. This study focused on evaluating two different support media for OM and N removal from an Upflow Anaerobic Sludge Blanket (UASB) reactor fed with swine wastewater. Maximum specific nitrification (MSNA) and denitrification (MSDA) activity test for both biofilm and suspended biomass were carried out using as supports: polyurethane foam (R1) and polyethylene rings (R2). The results showed that R2 system was more efficiently than R1, reaching OM removal of 77 ± 8% and N of 98 ± 4%, attributed to higher specific denitrifying activity recorded (5.3 ± 0.34 g NO_3_-N/g TVS∙h). Furthermore, 40 ± 5% of the initial N in the wastewater could have been transformed into molecular nitrogen through SND, of which only 10 ± 1% was volatilized. In this sense, MSDA tests indicated that suspended biomass was responsible for at least 70% of N removal and only 20% can be attributed to biofilm. SND could be confirmed with the analysis of microbial diversity, due to the presence of the genus *Pseudomonas* dominated the prokaryotic community of the system in 54.4%.

## Introduction

Nitrogen is an essential biological growth nutrient and one of the main constituents of all living organisms. However, its excessive presence should be avoided for the following reasons: (a) Nitrogen in reduced forms exerts oxygen demand in the receiving water body^1^, (b) Ammonia and nitrite are toxic for fish in concentrations higher than 0.045 and 0.20 mg/L, respectively^2^, (c) Wastewater with high nitrogen concentrations requires a great amount of chlorine for its disinfection^3^, and (d) Nitrite and nitrate in concentrations greater than 0.2 and 1.5 mg/L, respectively, jointly with phosphorus in concentrations greater than 0.10 mg/L may cause eutrophication of lakes and water bodies, which results in an uncontrolled growth of algae and other aquatic plants^4,5^. Nitrogen can appear in wastewater in different ionized forms: ammonium (NH_4_^+^) and ammonia (NH_3_^+^) that depending on the concentration, pH and temperature^6^.

Actually, different technologies have been proposed for the removal of nitrogen from water. These technologies include physicochemical processes such as ion exchange, adsorption, reverse osmosis and chemical processes such as active metal and catalytic methods^7–10^_._ However, these technologies are unfocused on the removal of high concentrations of ammonia and other N species, such as Jonoush et al.^11^ whose reported removal of nitrate in low concentrations (50 mg/L) using a non-noble Ni–Fe cathode. Various biological technologies have been developed for nitrogen removal, for example, (i) single reactor system for high ammonium removal over nitrite, best known as SHARON process, which is based on ammonium oxidation to nitrites of 50%, in low oxygen conditions (< 0.7 mg of O_2_/L); (ii) Anaerobic ammonium oxidation (ANAMMOX), where ammonium functions as electron donor and nitrite oxygen as electron receptor to obtain gaseous nitrogen; and (iii) Simultaneous nitrification–denitrification (SND), which is given by forming anoxic microzones in the interior of the bacterial consortia found in the aerobic reactor. The co-existence of aerobic and anoxic zones leads to the self-assembly of autotrophic nitrifying and heterotrophic denitrifying microorganisms in SND because of the OD distribution. Therefore, it is very important to perform anaerobic treatment of wastewater with high carbon content to promote subsequently in aerobic system a nitrification–denitrification and reduce competition for DO with heterotrophic microorganisms. A high C/N ratio influent can also negatively influence the abundance of nitrifying bacteria and nitrification process efficacy, due to the domination of heterotrophs. There are reports that total nitrogen (TN) removal rate reached 77% at a C/N ratio of 19.5, and a rate of 87% at a ratio of 7.7^12^. Therefore, SND has become in the most promising technology for removal of ammonium and other nitrogenous compounds in concentrations higher than 250 mg N/L, but deserves attention and more research is still necessary^13,14^.

In recent years, the combination of various treatment technologies has improved the removal of persistent pollutants. In this sense, the aerobic treatment system has been improved by combining with support material for growth of a wide variety of microorganisms in form of biofilm, which favors an easier control and is able to achieve a higher efficiency^15^. This process offers some distinct advantages over conventional activated sludge processes, including higher biomass concentrations, simplicity of operation and higher process stability, high effluent quality associated with suspended growth systems and the diffusional barriers of the biofilm, imply that the biomass is less susceptible to irreversible damage due to the potential occurrence of shock or toxic loadings^16^. In the system on a biofilm basis, the limited diffusion of oxygen and simultaneous diffusion of produced NOx as electron-acceptors inside the biofilm creates macro-anoxic areas within the same ecosystem. This can make it possible that simultaneous nitrification–denitrification can occur in the continuously aerated bioreactor independent of the solid retention time of suspended biomass^17^. There are a wide range of support materials, some of them may be organic or synthetic, for example: wood, gravel, rock and synthetic materials: ceramic, nylon, polyethylene and polyurethane^18^ Polyethylene and polyurethane are the most commonly used due to their surface area (200–1200 m^2^/m^3^) and the number of pores promote the adherence of bacterias^19^. Some advantages of these supports media have lower densities than water which can influence air flows, hydraulic speeds and impact on the mass and oxygen transfer^20^ as well as resistant, less volume requirement, no recycling or backwashing and no mechanical intervention in case of load fluctuations.

For the implementation of biological process understanding the microbial structure that conforms the biological system is needed for its proper function^1^. Nitrification is a biological aerobic process that oxidizes NH_4_^+^ to NO_2_^−^ with the help of ammonia oxidizing bacteria (AOB) followed by the conversion of nitrite to nitrate by oxidizing bacteria (NOB), both bacterial groups are called chemoautotrophic nitrifying bacteria^21^. *Nitrosomonas, Nitrosococcus* and *Nitrosospira* are the main bacteria reported for ammonia oxidation whereas *Nitrobacter, Nitrocystis* and *Nitrospira* have been reported for nitrite oxidation^22^. Denitrification is a non-assimilative reduction process of oxidized nitrogen forms (NO_2_^−^ and NO_3_^−^) to molecular nitrogen under anoxic conditions. This process is carried out by heterotrophic bacteria using organic carbon source for their metabolism^22^. Denitrification may be carried out by diverse groups of bacteria, but they are generally heterotrophic microorganisms (*Phylum, Proteobacteria, Firmicutes, Thiobacillus versutus*, etc.) and less frequently by autotroph organisms (*Thiobacillus denitrificans* and *Mocrococus denitrificans*)^23^. Examples of the different bacterial population diversity in aerobic system with support media, both in suspended biomass and biofilms can be *Enterobacter cloacae*, *Vibrio diabolicus*, *Bacillus* and *Pseudomonas stutzeri.* The last bacteria have been isolated from swine manure and has the ability to remove nitrate and nitrite and capability to reduce ammonium to nitrogen gas under aerobic conditions^24^, indicating that this strain can perform heterotrophic nitrification and aerobic denitrification^25^ Although some researchers have studied SND in biofilms from a metabolic and microbial population point of view^26^, there is little information on systems with suspended and fixed biomass that have simultaneous nitrification–denitrification.

In this context, the objective of this study is to assess nitrogen and organic matter removal from two aerobic fixed-film systems with polyurethane foam (R1) and polyethylene rings (R2). Additionally, a microbiological analysis of the most efficient pack-bed biofilm system is performed to determine the main microorganisms involved in nitrogen removal process.

## Materials and methods

### Inoculum

The biomass used for the experiments was obtained from an aerated lagoon as part of the process of a wastewater treatment plant located southward from Cd. Obregón, Sonora, in northwest Mexico. The reactors were inoculated with the support media previously placed in contact for one week with 1 L of aerobic biomass 20.4 g/L of total solids (TS) and 7.66 g/L of Total Volatile Solids (TVS).

### Operation of the aerobic systems

**Figure 1:**
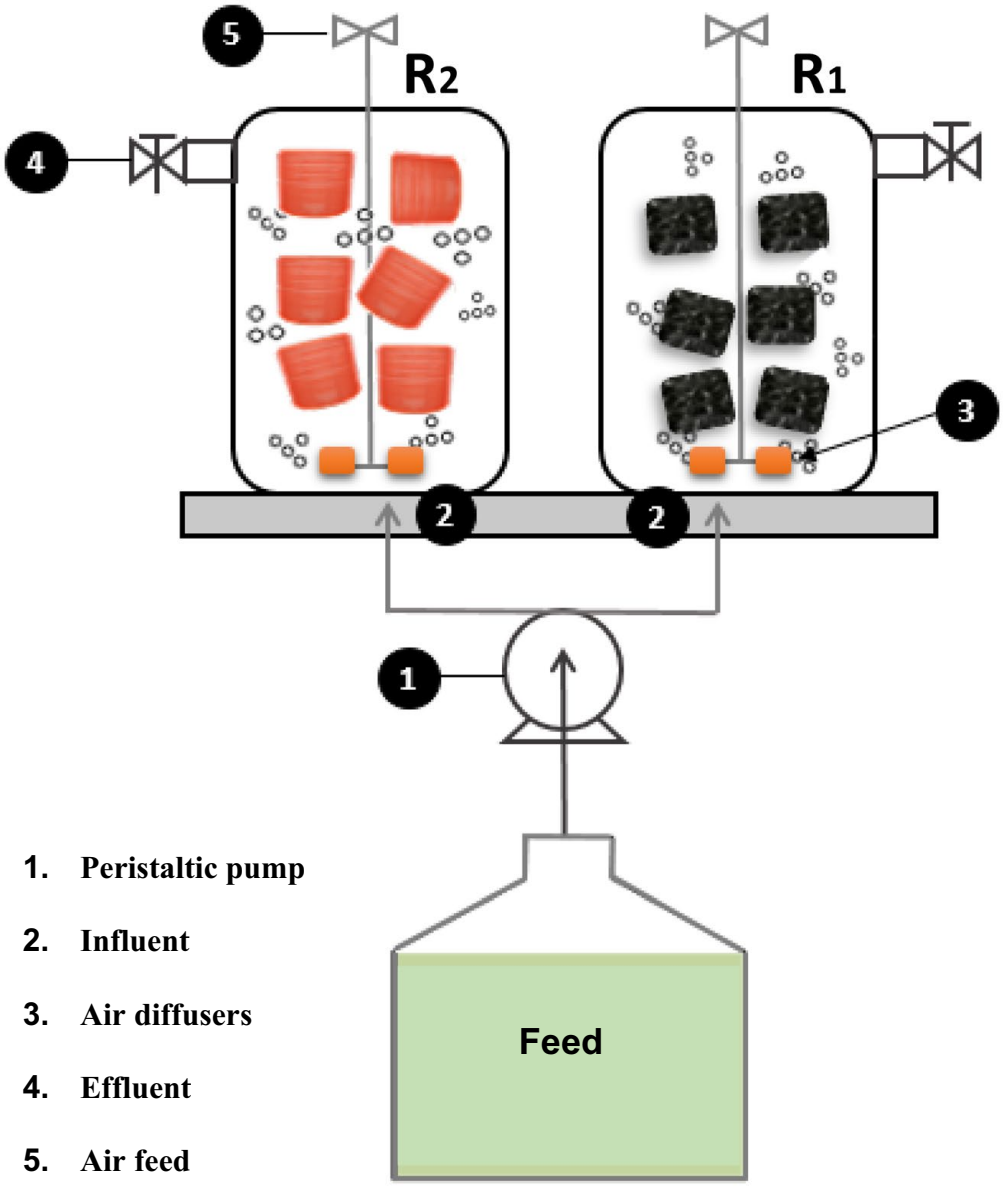
Schematic diagram of aerobic Fixed-film systems.

The two aerobic fixed-film reactors, R1 (filled with polyurethane foam) and R2 (filled with polyethylene rings) had a capacity of 0.9 L each one and operated continuously for 330 days with hydraulic retention time (HRT) from 0.4 to 0.5 days and an average dissolved oxygen (DO) concentration of 3.35 mg/L. Buttom up aeration was performed in the reactors through the mammoth pump. Figure 1 shows the schematic diagram of the systems. Table 1 shows the characteristics of the support materials.

**Table 1:** Packing media characteristics.

Parameter	R1	R2
Material	Polyurethane	Polyethylene
Dimensions	Width: 0.5 cm; Height: 0.5 cm	Diameter: 3 cm; Height: 3 cm
Shape	Rectangular	Cylindrical
Percentage in system (%)	20	20
Support mass (unit)	1.2	6
Density (g/L)	7.3	77.8
Superficial Area (m^2^/g)	23.5*	6.1**

*Sahariah et al.^38^, **Silva et al.^51^.

### Influent characteristics

The fixed-film reactors were fed with the effluent of an UASB that treated swine wastewater coming from a farm with a maternity production process. Table 2 shows the physicochemical characteristics of the two fixed-film system feed evaluated, which maintained an organic load rate (OLR) of 0.6 ± 0.3 kg of chemical oxygen demand (COD)/m^3^day.

**Table 2:** Physicochemical characteristics of packed systems influent.

Parameter*	Concentration (mg/L)
Chemical oxygen demand (COD)	300 ± 100
Nitrate (NO_3_^−^ N)	5 ± 4
Nitrite (NO_2_^−^ N)	5 ± 4
Ammonium (NH_4_^+^-N)	100 ± 20
Total solids (TS)	650 ± 150
Total volatile solids (TVS)	200 ± 100

*The parameters were quantified according to the techniques described in the analytical methods section.

### Analytical methods

In the influent and effluent of the fixed-film reactors, the following analytical techniques were performed: organic matter chemical oxygen demand (COD), total solids (TS), Total Volatile Solids (TVS), nitrates (NO_3_^−^ N), nitrites (NO_2_^−^ N) and ammonium (NH_4_^+^-N) according to that established in the American Public Health Association^27^.

### Nitrifying and denitrifying activities

To know the nitrifying and denitrifying activities of the systems evaluated, discontinuous assays were performed based on the proposed methodology by Bassin et al.^28^. In the nitrifying activity assay, R1 and R2 were evaluated (1) with suspended biomass + biofilm in the support material and (2) only with suspended biomass (without support material). The nitrifying activity was performed discontinuously, stopping system feed, followed by injecting a stock solution of 100 mg NH_4_^+^-N/L. The samples were collected from the supernatant of each reactor every hour for 10 h and subsequently every two hours with a total of 36 h. To know the nitrifying activity, soluble nitrogen forms (NH_4_^+^-N, NO_2_^−^ N and NO_3_^−^ N) were determined in each established time. After determining NH_4_^+^-N removal at the final sampling time, its volumetric removal rate (VRR) was calculated according to Eq. (1):

1 $${\text{VRR }}\;\left( {{\text{mg}}\;NH_{4}^{ + } - {\text{N}}/{\text{L}} \cdot {\text{h}}} \right){ = }\frac{{NH_{4}^{ + } - {\text{N }}_{{\text{initial }}} - {\text{N}} - NH_{{4 {\text{final}}}}^{ + } \;\left( {{\text{mg}}/{\text{L}}} \right)}}{Time\; \left( h \right)}$$

The result of the maximum specific nitrifying activity (MSNA) in mg NH_4_^+^-N/g TVS∙h was obtained from dividing the volumetric removal rate (VRR) of nitrogen in the form of ammonium by the TVS of suspended and fixed biomass concentration (Eq. 2):

2 $$MSNA \left(mg {NH}_{4}^{+}-\frac{\mathrm{N}}{g}TVS\cdot h\right)=\frac{\mathrm{VRR }(\mathrm{mg }{NH}_{4}^{+ }-\mathrm{N}/\mathrm{L}\cdot \mathrm{h})}{ (\mathrm{g }/\mathrm{L})}$$

With respect to the denitrifying activity, it was developed similarly to the nitrifying one, also performed in the two aerobic systems discontinuously stopping their feed. During the maximum specific denitrifying activity (MSDA) the R1 and R2 systems were injected with a concentrated stock solution of NaNO_3_, and C_2_H_3_NaO_2._ As a result, concentrations at time zero 82 ± 0.17 and 77.71 ± 2.02 mg of NO_3_^−^ N/L and 261.42 ± 8.75 and 250 ± 5.41 mg COD/L were obtained for R1 and R2, respectively, with the purpose of reaching a theoretical value of 80 mg of NO_3_^−^ N/L and 250 mg of COD/L, simulating the conditions of the influent in the continuous systems.

Times for sampling in this experiment were equal to those of the nitrifying activity. After determining nitrogen concentrations in nitrates in the final sampling time, the VRR of NO_3_^−^ N was calculated and obtained by dividing nitrogen concentration in the form of removed nitrate in mg/L by the time in hours (Eq. 3):

3 $$\mathrm{VRR }\left(\mathrm{mg }{NO}_{3}^{-}-\frac{\mathrm{N}}{\mathrm{L}}\cdot \mathrm{h}\right)=\frac{{{NO}_{3}^{-}-\mathrm{N }}_{initial }- {{NO}_{3}^{-}-\mathrm{N}}_{final}(\mathrm{mg}/\mathrm{L})}{Time (h)}$$

Subsequently, the MSDA was obtained from dividing nitrogen VRR in nitrates by the TVS of biomass concentration in the systems (Eq. 4):

4 $$MSDA \left(mg {NO}_{3}^{- }-\frac{\mathrm{N}}{g}TVS\cdot h\right)=\frac{\mathrm{VRR }(\mathrm{mg }{NO}_{3}^{-} -\mathrm{N}/\mathrm{L}\cdot \mathrm{h})}{ (\mathrm{g}/\mathrm{L})}$$

### Extraction, amplification and DNA analyses in supports

Biofilm volumes of 1.5 mL were taken in sterile conical microtubes and centrifuged at 5000 rpm for 15 min. The supernatant of each microtube was discarded, and the final pellet was used in the nucleic acid extraction. The total DNA extraction was performed starting from 0.1 to 0.12 g of pellets with the DNeasy PowerSoil Kit (Qiagen, Hiden, DE) according to the manufacturer’s instructions. Subsequently to the extraction, DNA was quaintified in a fluorometer Qubit 3.0 (ThermoFisher Scientific, Waltham, MA, USA), preserving the samples at -20 °C up to their subsequent processing. The diversity of bacteria and archaea was determined by amplifying the V4 16S rRNA region with the specific primers 515F (5’-GTGCCAGCMGCCGCGGTAA-3’) and 806R (5’-GGACTACHVGGGTWTCTAAT-3’). The endpoint polymerase chain reactions (PCR) were performed in a total volume of 25 μL, whose concentration for each reaction consisted of: ~ 10 ng DNA, 1X PCR buffer (free of Mg^2+^), 0.4 μM of each oligonucleotide primer, 800 μM of deoxynucleoside triphosphate (dNTP) mix (dATP, dCTP, dTTP and dGTP), 5% of dimethylsulfoxide (DMSO), 1.5 mM of MgCl_2_ and 1 U of DNA Taq polymerase ExTaKaRa Taq (Takara Bio Inc. Kusatsu, Shiga, JP). The amplification protocole consisted of an initial DNA denaturalization at 95 °C for 3 min, followed by 35 denaturalization cycles (95 °C, 30 s), hybridation (52 °C, 40 s) and extension (72 °C, 90 s), with a final extension at 72 °C for 10 min. The amplicons obtained were purified with magnetic pearls using the Agencourt AMPure XP PCR Purification System (Beckman Coulter, Brea, CA, USA) kit and shipped for sequencing in the plataform Illumina MiSeq (Yale University, USA). The sequences obtained were analyzed with the platform QIIME2 (https://qiime2.org)^29^. The amplicon sequence variant (ASV) was taxonomically classified using Silva (https://www.arb-silva.de/) database. The charts and relative abundance of the main microbial groups were performed with the program ‘phyloseq’^30^ in the RStudio (1.2.5042) environment of the R (The R Core Team 2012) platform.

### Statistical analyses

The results were expressed as average standard deviation (SD) ±. Data were analyzed using a two-way analysis of variance (ANOVA) with the Minitab (Version 17.0) software. When ANOVA identified the differences among groups, multiple comparison of means was performed using Tukey’s honest with a level of significance of p < 0.05.

## Results and discussion

### Aerobic fixed-film system performance

**Figure 2:**
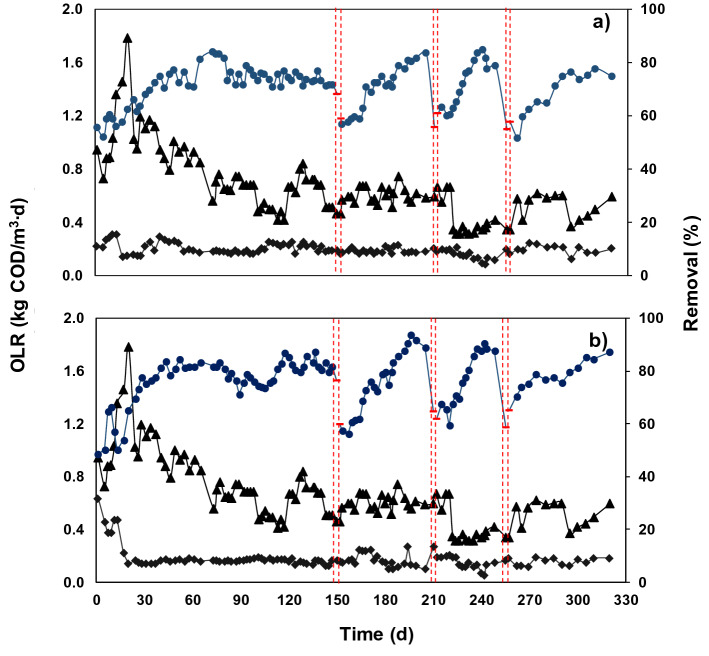
Removal efficiency of organic load rate (OLR) (kg COD/m^3^∙d) with UASB effluent. Where: (**a**) is R1 with polyurethane foam and (**b**) is R2 with polyethylene rings. (filled circle) Removal of chemical oxygen demand (COD) percentage; (filled triangle) influent and (filled diamond) effluent. Lines show days in which nitrifying and denitrifying activities were performed in both discontinuous reactors.

Organic matter removal was evaluated by measuring organic load rate both in the influent and effluent of the systems (Fig. 2). The influent concentration was maintained in 300 ± 100 mg COD/L and both R1 and R2 indicated a similar COD removal of organic matter of 72 ± 7 and 77 ± 8%, respectively. It is worth to mention that the system feed was stopped to perform the nitrifying activity tests with suspended biomass biofilm (day 150) only with suspended biomass (day 200) and denitrifying activity (day 250). Additionally, suspended biomass and biofilm samples were taken subsequent to each assay to quantify the volatile suspended solids (VSS). Thus, destabilization in R1 and R2 was observed in these days causing decreases in removal efficiency (Fig. 2). The statistical analyses showed that no significant difference was observed in organic matter removal between R1 and R2 (p ≤ 0.05), reaching a stationary state after the first 50 days of operation.

Similar results have been reached by mobile packed-bed bioreactors coupled to membrane bioreactors (MBBR-MBR) fed with domestic wastewater with a COD concentration of 185.80 ± 45.8 mg/L and operating with a hydraulic retention time (HRT) of 0.5 days. These packed-bed systems with a commercial Kaldnes type “K_1_”, in a filling ratio of 35% of total volume, showed global COD removal efficiency of 83 ± 2.11%^31^. Mazioti et al.^32^ reported COD removal efficiency of 86.6% when they operated a MBBR system of 4.5 L useful volume with domestic wastewater and influent concentration of 270 mg COD/L, operated with HRT of 1.1 day, packed with the commercial AnoxKaldnes type “k3” support material in a filling volume ratio of 30%. Boutet et al.^33^ obtained COD removal efficiencies of 47%, operating packed-bed systems with inert material “BIONEST” using municipal wastewater with an average concentration of 457 mg/L COD and HRT of 0.5 day.

In general, the systems assessed in this study reached similar or higher efficiency to those reported by other authors, which not only depended on variables, such as filling material percentage, COD concentration in the influent or type of supporting material but also on biomass concentration developed in the system interior. The biomass developed on the material surface was 18 ± 5 and 21 ± 3 g TVS/L of R1 and R_2_, respectively. This biofilm possibly gives rise to different oxygen concentration gradients towards the interior of these materials. The differences in oxygen concentration in the system could favor the appearance of anoxic zones in the deepest areas of the biofilm where oxygen cannot easily penetrate. The previous allows heterotroph organisms to assimilate organic carbon for their metabolism and growth, giving rise to denitrification processes and favoring removal of organic matter in the form of COD^13,34^.

**Figure 3:**
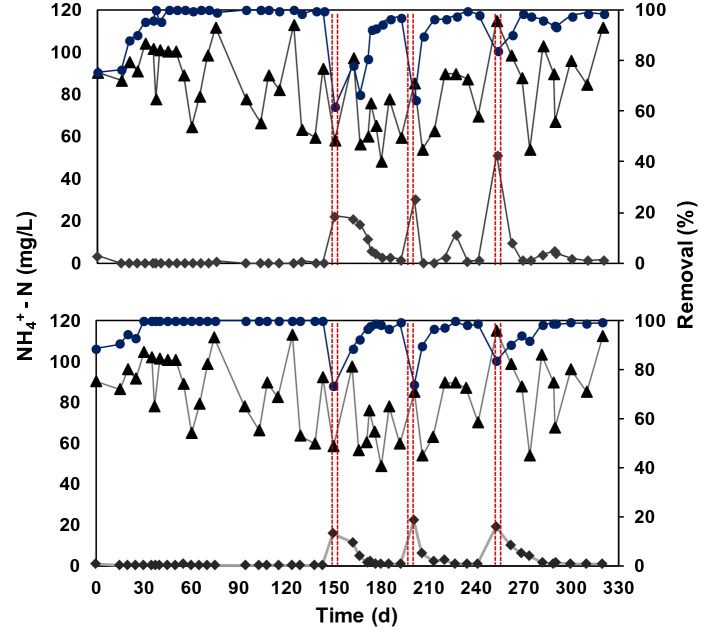
Removal efficiency of NH_4_^+^-N with UASB effluent. Where (**a**) is R1 polyurethane foam and (**b**) is R2 polyethylene rings. (filled circle) Chemical Oxygen Demand (COD) removal percentage; (filled triangle) influent and (fille diaamond) effluent. Lines show days in which the nitrifying and denitrifying activities were performed discontinuously in both reactors.

Figure 3 shows the fixed-film system performance for NH_4_^+^-N removal. It is worth to point out that this performance represents the system behavior when fed with the UASB effluent, while the nitrifying and denitrifying activities were performed with synthetic solutions as the methodology indicated. The influent concentration was maintained in 100 ± 35 mg NH_4_^+^-N/L for 330 days of operation of the systems R1 and R2 showing a complete removal efficiency of NH_4_^+^-N (99.9%). According with the low C/N ratio in the influent of R1 and R2 (⁓7) this may have been a positive effect on the abundance of nitrifying and denitrifying bacteria and promote a SND process.

Aditionally, Lo et al.^35^ concluded that high efficiency is possible thanks to the nitrifying activity carried out in aerobic systems with support. The nitrifying activity is performed both because of the suspended biomass within the systems and fixed biomass adhered to the supports. However, Bassin et al.^28^ demonstrated that suspended biomass plays the most important role in ammonium removal process, showing a nitrifying activity relatively greater compared to biofilm, which plays the main role in the denitrifying process. Additionally, total nitrogen removal may be achieved indirectly as a result of both SND process and nitrogen assimilation by the heterotroph organisms for new cell formation. Lo et al.^35^ reported that approximately 34% of total initial nitrogen was used for biomass formation where a SND process was performed in a hybrid biofilm system with an HRT of eight hours. This result implies a process where the greatest part of nitrogen is removed by a SND process, but with a yield in biomass formation. Coupled to this process, bacteria involved in SND, such as *Pseudomonas*, have low replication times of up to 30 min^34^ which is why nitrogen assimilation through these bacteria possibly plays an important role in R1 and R2 performance.

Matsumoto et al.^36^ and Wu et al.^37^ observed SND process in biofilm systems with inert materials, such as plastic and ceramic membranes, evidencing these processes by the presence of AOB and NOB bacteria in the internal zone of the biofilm and heterotroph bacteria in the same biofilm surface. In this sense, according to Bassin et al.^28^, up to 20% of NH_4_^+^-N removal might be attributed to biofilm while the suspended biomass contributed up to 70% of this process. Lastly, Fig. 3 also shows that NH_4_^+^-N removal efficiency reached by each system were stable and without significant differences between them, according to statistical analyses (p < 0.05).

Sahariah et al.^38^ operated a sequential mobile packed-bed bioreactor with polymer foam support with a filling volume of 15.7% and fed with a concentration of 125 mg NH_4_^+^-N/L. The reported systems showed removal efficiency of 68% of NH_4_^+^-N, a lower value than that obtained in this study. On the other hand, Bassin et al.^28^ operated two mobile packed-bed bioreactors of 1 L of useful volume, one packed with the commercial synthetic support “Kaldnes K1” and the other one with “MutagBiochip” with the filling volume of 50%, operated with HRT of 0.5 days and fed with a concentration of 100 mg NH_4_^+^-N/L. The authors achieved NH_4_^+^-N removal efficiency higher than 90%. It should be mentioned that the support materials evaluated in this study occupied a filling volume with a range from 15 to 50%, reaching NH_4_^+^-N removal efficiency of > 99%. In this sense, the systems evaluated in this study demonstrated much higher efficiency with respect to similar systems. As mentioned previously, this excellent performance could have been due to biofilm presence (18 ± 5 and 21 ± 3 g TVS/L for R1 and R2, respectively), which was measured at the end of the assays. Ødegaard et al.^39^ and Bassin et al.^28^ suggested that the quantity of adhered biomass to a support medium not onl1y depends on the superficial area but also its form or material configuration. These findings indicate that supports, as Mutag Biochip that has the form of a satellite dish, are frequently subjected to attrition forces due to the intense contact with the surrounding liquid, favoring biofilm detachment and the quantity of adhered solids. Whereas the types of support with cylindrical shape or rings favor biofilm accumulation.

### Nitrifying and denitrifying activity

**Figure 4:**
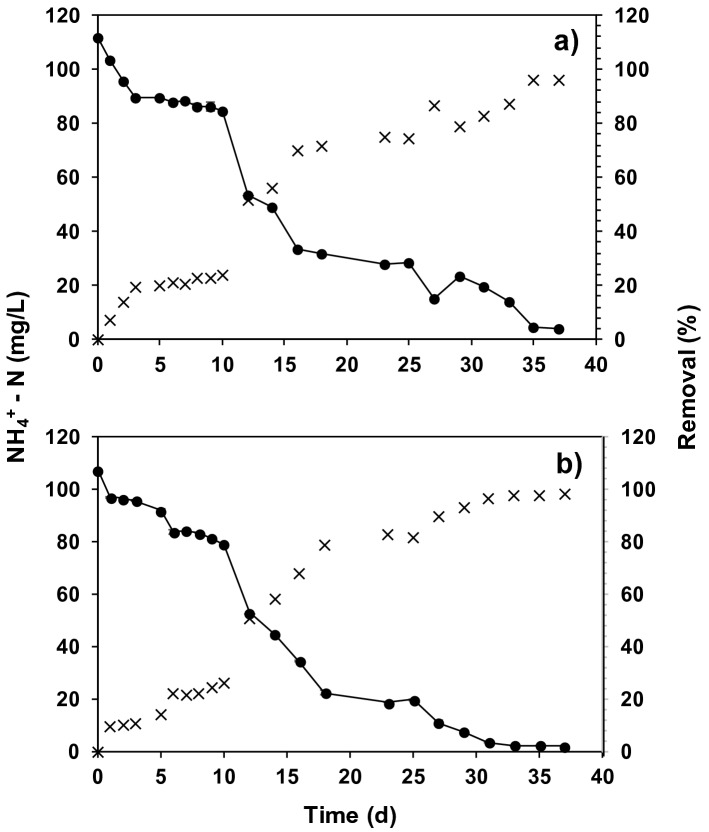
Behavior of NH_4_^+^-N concentration in suspended and fixed biomass assays (SB + FB), where: (**a**) R1 is polyurethane foam (**b**) R2 is polyethylene rings. (X**)** removal efficiency follow-up of  mg NH_4_^+^-N/L  (filled circle).

The nitrifying and denitrifying activity of the systems were assessed in a period of 36 h for R_1_ and R_2_. The systems operated continuously and for these assays they were set in batch mode, stopping feed flux. Figure 4 shows ammonium removal during the MSNA assays. The NH_4_^+^ removal was 20 ± 5% for both systems at hour 10 of the assay; five hours later the systems reached two-fold removal. Starting from hour 10, removal started to increase significantly until it reached 90 ± 6 and 98 ± 4% of NH_4_^+^-N removal for R_1_ and R_2_, respectively (Fig. 4). This result could indicate an adaptation process of the microorganisms in the systems when they went from operating continuously to discontinously. Although it did not inhibit the nitrifying process, it made it slower.

Despite the behavior was similar for both assays (R1 and R2), the R_2_ system reached greater NH_4_^+^-N removal, whereas R1 showed a slightly lower removal though not significant according to the statistical analyses performed (p > 0.05). Additionally, at the end of the assay, loss of nitrogen was evident in both systems, but it was not found in any of the determined soluble forms, approximately 60% and 65% for R1 and R2, respectively. Presumably, these non-quantified nitrogen percentages have been converted to molecular nitrogen by means of SND. Garzón-Zuñiga et al.^13^ explained that aeration systems with fixed biomass in support materials are capable of developing denitrifying processes starting from heterotroph bacteria that achieve growing in anoxic environments. On their part, Lo et al.^35^ studied nitrogen transformation in the form of ammonium to nitrogen, gas in a hybrid biofilm system. The results showed that approximately 60% of soluble nitrogen was converted to nitrogen gas by a SND process. On the other hand, some *Pseudomonas* species have been reported capable of reducing nitrogen compunds in denitrification process^40^. Zhang et al.^25^ isolated a *Pseudomonas stutzeri* YZN-001 from swine manure effluent and evaluated the reduction of all nitrogen species. For example, this strain had the capability to remove 275.08 mg/L of nitrate and 171.40 mg/L nitrite under aerobic conditions. Moreover, 39% of removed ammonium was completely oxidised to nitrogen gas, indicating that this strain could achieve heterotrophic nitrification and aerobic denitrification.

Table 3 shows the results of MSNA, as well as those reported by different authors, where the obtained values are found within the range bibliographically reported for systems operated under similar conditions. The results allow observing the importance of suspended biomass in MSNA: 3.13 and 2.05 mg NH_4_^+^-N/g TVS∙h for R_1_ and R_2_ respectively, even higher including that reached by the systems with both biomass types (suspended and fixed): 0.352 and 0.253 mg NH_4_^+^-N/g TVS∙h for R1 and R2, respectively. Lo et al.^35^ observed that in a hybrid biofilm system, nitrification was produced mainly in suspended biomass while biofilm played the main role in denitrification. In this manner, biofilm and suspended sludge interaction in the same reactor gave as a result a better general yield in nitrogen removal by a SND. The previous information may be observed in the nitrifying assays (Table 3). On the other hand, Mašić and Eberl^41^ found evidence through mathematical models that suspended biomass contributes in a more important manner to ammonium removal in biofilm systems. However, nitrifying activity is not considered frequently in the suspended biomass, assuming that nitrification only takes place in the biofilm^42^.

**Table 3:** Specific nitrifying activity obtained in different laboratory-scale studies.

	Bassin et al.^28^	Salvetti et al.^52^	Bassin et al.^53^	Reif-Lopez^54^	Lu et al.^55^	This study	
Type system	MBBR	MBBR	SBR	MBR	SBBR	Packed-bed	
Material	Kaldnes K1 y MutagBiochip	KMT	NR	Membrane Zenon ZW-10	Polyurethane	Polyurethane	Polyethylene
Ammonium	0.2	1.96	0.2	0.04–0.08	NR	0.18	
OLR	0.82–3.2	1.28	0.9	0.45–0.9	NR	0.75	
HRT	12–3.1	0.3–0.6	5.2	24–12.1	12	12	
TVS	4.9–5.52	6.67	10.0–14.2	0.5–2	NR	15.79	20.44
MSNA	1.2–5.6^a^	15-44^a^	1.2–5.6^a^ 9.5-18^b^	0.12–0.16^a^	7.0-22^a^	0.35^a^ 3.13^b^	0.25^a^ 2.05^b^

^a^Fixed biomass, ^b^Suspended biomass. Where: Maximum specific nitrifying activity (MSNA) is expressed in mg NH_4_^+^-N/g TVS∙h; Volumetric organic load rate (OLR): kg COD∙m^3^/d;

Ammonium load: kg NH_4_^+^-N/m^3^; Hydraulic retention time (HRT): h; Total volatile solids (TVS): g/L. *NR* no reported.

On the one hand, MSDA was 4.64 ± 0.13 and 5.3 ± 0.34 mg of NO_3_^−^ N/g TVS∙h, for R1 and R2, respectively, results that are found within the range reported for biological nitrogen removal (BNR) systems. On the other hand, the determined MSDA agreed with that reported for the SND processes (1.6–30 mg of NO_3_^−^ N/g TVS h) for BNR systems inoculated with aerobic biomass and fed with real wastewater. Whereas lower MSDA values were reported for conventional denitrification routes and ANAMMOX (0.5–1.56 mg of NO_3_^−^ N/g TVS h)^43^. In the case of R1 and R2, nitrification and denitrification metabolism activation is performed in the same system simultaneously SND and not in different or sequential reactors as conventionally reported^31^. The previous is due to the presence of anoxic microzones in the aerobic system, given as a result dissolved oxygen gradients that limit their diffusion through the systems^43^.

In this sense, the main explanation for SND is because denitrification organisms may exist both in the biofilm and suspended biomass of the system. Additionally, the existence of facultative microorganisms has been proven that use NH_4_^+^-N as electron donor and NO_2_^−^ N as electron receptor, producing N_2_ and NO_X_ in SND^13^.

In the case of MSDA, polyethylene rings showed superiority over polyurethane foam, which was directly related to the amount of developed biofilm. Thus, polyethylene rings were selected as the most efficient support material in NH_4_^+^-N removal. Additionally, the statistical analyses indicated a significant and higher difference (p ≤ 0.05) in the denitrifying activity, where the rings could favor SDN alternate routes because of factors, such as configuration and material type that could create better conditions to form anoxic zones where the denitrification process mainly takes place.

**Figure 5:**
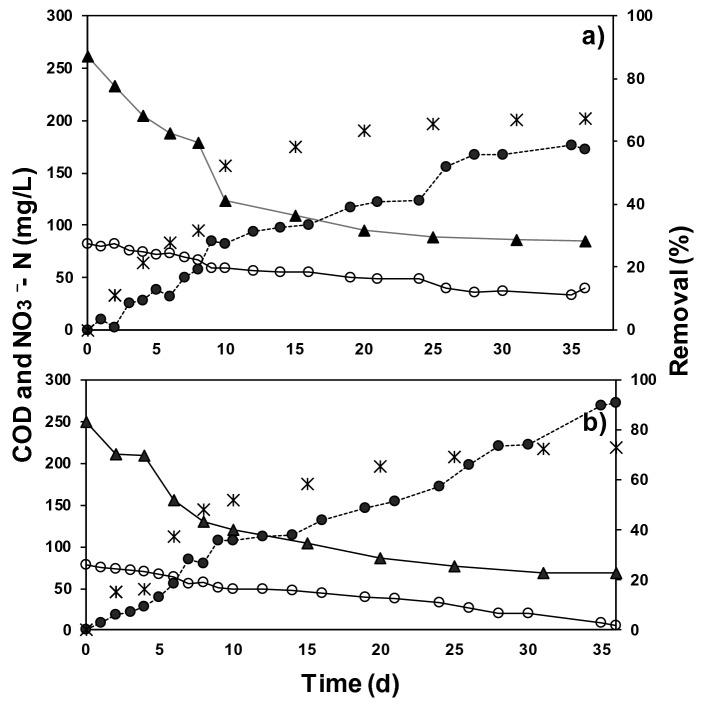
Time chart of consumption behavior NO_3_^−^ N and chemical oxygen demand (COD) during the maximum specific denitrification activity (MSDA) assays for (**a**) R1 and (**b**) R2. Where (filled circle) is the concentration of NO_3_^−^ N; (o) is NO_3_^−^ N removal percentage; (filled triangle) COD concentration (mg/L) and (ӿ) is COD removal in the systems.

Figure 5 shows the results obtained from monitoring NO_3_^−^ N behavior, organic matter in the form of COD and removal efficiency for the MSDA assays. Differently from MSNA, R1 and R2 had a different behavior, of which system R2 was the most efficient by removing 91 ± 2.24% of NO_3_^−^ N and 67.86 ± 0.4% of COD, whereas system R1 removed 52.32 ± 0.6% of NO_3_^−^ N and 57.42 ± 1.24% of COD. The results indicated that 2.54 g COD/g of NO_3_^−^ N reduced were used, which correspond to the organic requirements reported by Chatterjee et al.^44^ for heterotrophic denitrification (2.86 g COD/g of NO_3_^−^ N removed), and more specifically 2.08 g COD/g of NO_3_^−^ N reduced when using C_2_H_3_NaO_2_ as a carbon source^12^.

### Balance of nitrogen species

The result of the nitrogen forms measured in the effluents R1 and R2_,_ evidenced a nitrogen concentration that could not be quantified (~ 40 ± 5%) with respect to NH_4_^+^-N measured in the influent. The material balance indicated that it was quantified in the effluents of R1 and R_2_: 55 ± 11% and 54 ± 10% in the form of NO_3_^−^ N; 2.58 ± 2 and 3.4 ± 2.5% in NO_2_^−^ N and 3.03 ± 4.02% and 5.07 ± 6.84% as NH_4_^+^-N. Based on these results, the operated systems might have shown a SND process.

According to Matsumoto et al.^36^ SND is associated to reactors that have suspended biomass and biofilm and show nitrogen loss in the effluent. Anoxia conditions activate denitrifying metabolism, which are given by the anoxic microzones in the interior of the biofilm bacterial consortium. In these microzones, oxygen cannot penetrate, but the NOx generated by the nitrifying bacteria can. According to Garzón-Zúñiga^13^, the nitrates produced by the nitrifying bacteria in the superficial layers of the biofilm may penetrate toward the deepest layers by one concentration gradient. When they penetrate toward these deepest layers where oxygen concentration is very low or null, the denitrifying bacteria use nitrites and nitrates as receptor and transform them into molecular nitrogen (N_2_), which escapes from the system with gaseous effluent, making it possible to be counted in soluble form.

The previous information agrees with the volatilization assays that were performed additionally where a loss of NH_4_^+^-N to the environment in gaseous form was determined 10 ± 1%. It is worth to mention that some authors also reported loss by Stripping of 8–15%^11,37^. In this sense, Garzón-Zúñiga et al.^13^ found that in a packed-bed biofilter with organic material, nitrogen loss was performed by biologic sorption, filtration and assimilation mechanisms. These authors reported that from a total NH_4_^+^-N found in the influent, 10% oxidized NO_2_^−^ N and another 10% to NO_3_^-^ N, 40% was lost during the SND processes, 10% volatilized, 6% was retained in the system and 3.5% was found as residual NH_4_^+^-N. Zhao et al.^26^ also reported SND processes in packed-bed systems, examining the combination of different support media, such as grapefruit skin and several conventional plastics as polyurethane, SPR-1 suspension and elastic filling TA-II. The results showed that by combining these materials efficient SND processes could be achieved with total ammonium and nitrogen removal of 96.8 ± 4.0% and 78.9 ± 9.5%, respectively. Additionally, the microbial analysis evidenced dominant genera of *Thiothrix, Gemmata* and *Comanonadaceae*, which indicated a heterotroph nitrification—same which favored the SND process. Furthermore, Walters et al.^45^ operated a batch system with suspended biomass and biofilm adhered to a biodegradable support medium. The results and experiments of these authors clearly indicated that nitrification may be achieved in suspended biomass while denitrification is performed at the interior of the support structure pores.

### Microbial community analyses

The microbial community found in the biofilms of polyethylene rings was analyzed. This system was selected for analysis to show better performance as to the capacity of nitrogen and organic matter removal besides a larger concentration with respect to polyurethane foam. This study was performed through the analysis of the 16S rRNA fragments. A taxonomic classification of the total microbial community diversity was performed, which highlighted the microorganisms obtained at the level of phylum and genus. The bacterial abundance obtained from the sample was 99%. Table 4 shows that *Proteobacteria* was the dominant phylum that biofilm rings conformed, followed by *Bacteroidetes* and *Firmicutes* that are common in swine wastewater^46^. This result agrees with that reported by Alzate^47^, who mentioned that typical microbiology of aerobic systems with activated sludge are composed approximately of 95% bacteria. On the other hand, a certain abundance of archaea was observed, which was not significant (~ 1%).

**Table 4:** Relative abundance of different edges identified in biofilm of polyethylene rings.

Dominium	Phylum	Relative abundance (%)
Bacteria	Proteobacteria	56.10
	Bacteroidetes	24.54
	Firmicutes	9.59
	Tenericutes	3.54
	Spirochaetes	2.43
	Fibrobacteres	0.73
	Kiritimatiellaeota	0.50
	Verrucomicrobia	0.36
	Epsilonbacteraeota	0.34
	Cloacimonetes	0.32
	Actinobacteria	0.20
	Otros	0.28
Archaea	Euryarchaeota	0.91
	Nanoarchaeaeota	0.17

Within these phyla, the presence of *Pseudomonas* was detected in the biofilm rings. This bacterial genus is associated to denitrification processes in the presence of aeration^48,49^. Zhang et al.^25^ identified *P. stutzeri* in swine wastewater. These authors concluded that this type of *Pseudomonas* may transform not only nitrate and nitrite but also ammonium with the capacity of a complete removal up to 200 mg/L of NO_3_^−^ N and 170 mg/L of NO_2_^−^ N in aerobic conditions. They also observed NH_4_^+^-N removal of approximately 95% through a denitrification process and from this one 39% of NH_4_^+^-N removed was oxidized completely to gaseous nitrogen in a total of 18 h. This result indicated that the strain has capacities for heterotrophic nitrification and aerobic denitrification with the notable capacity of removing nitrogen efficiently in the form of ammonium. This percentage agrees including with 40% to the nitrogen not found in the effluent systems of this study in any of its soluble forms.

On the other hand, the presence of *Clostridium* (2.43%) in the biofilm rings would indicate nitrification processes^23^. Interestingly, bacteria of the type *Nitrosomonas* and *Nitrobacter* -responsible for nitrification in aerobic conditions were not found in the taxonomic analysis despite having obtained a removal efficiency of NH_4_^+^ greater than 95% in system R_2_. It should be highlighted that according to the MSNA assays, only may 20% of NH_4_^+^-N removal be attributed to biofilm, whereas 80% of this process would have been performed by suspended biomass, which was not microbiologically analyzed.

**Figure 6:**
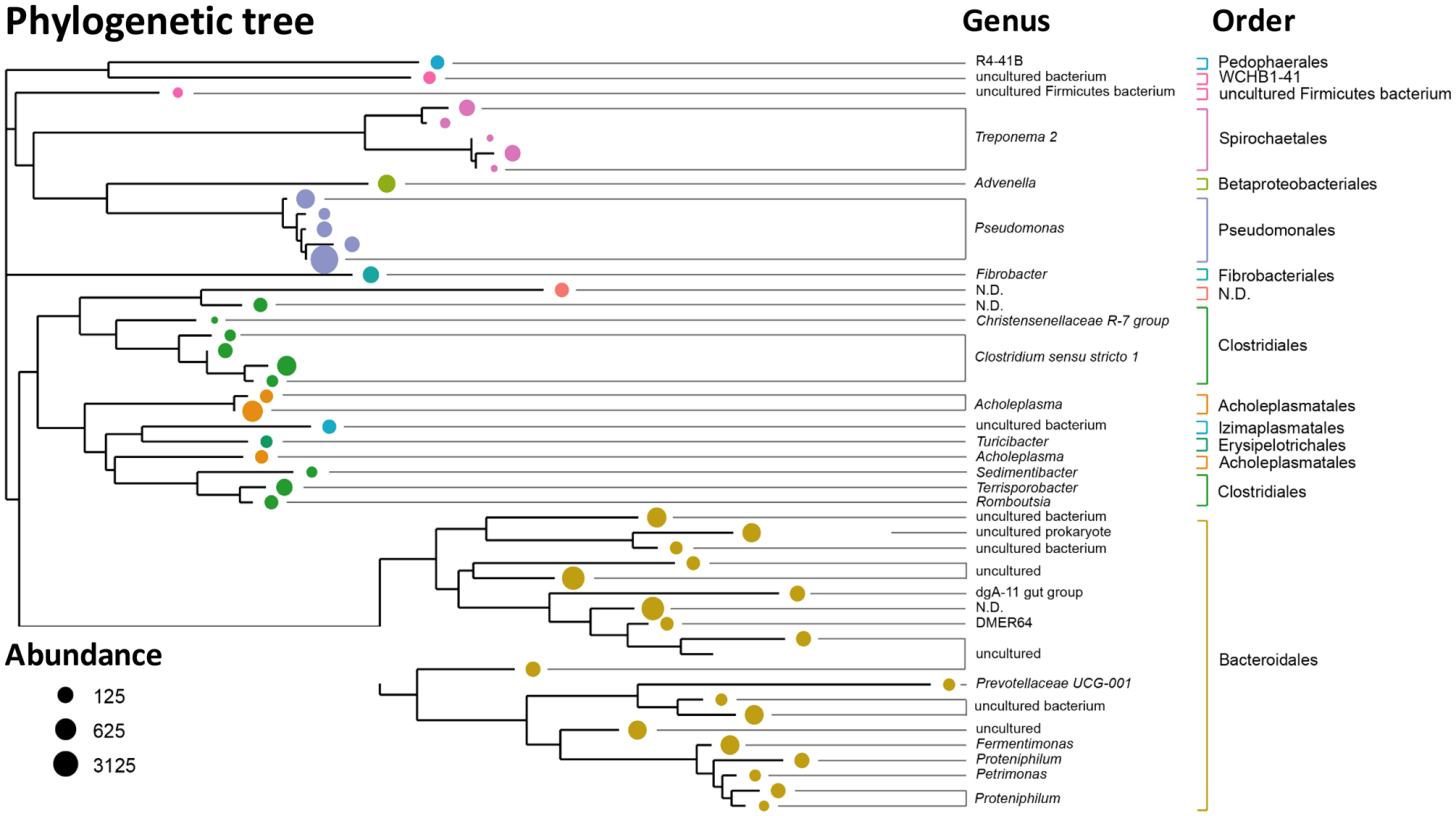
Phylogenetic tree of the most abundant bacteria presents in the R2 biofilm.

Figure 6 shows a phylogenetic tree of the 50 most abundant bacteria found in biofilm polyethylene rings. The circles of different size correspond to abundance in readings of each microorganism, while the color indicates the order to which the genus represented in the tree belong. Finally, the bacteria not classified at the level of order and/or genus are indicated.

The following bacteria at the level of order and by abundance are: Pseudomonadales (54.81%), Bacteroidales (24.17%), Clostridiales (8.59%), Acholeplasmatales (3.01%), Spirochaetales (2.01%). The rest of the organisms that appear in Fig. 6 were found in a percentage lower than 1%. In contrast with the results mentioned, Nascimento et al.^50^ reported that bacteria of the order Clostridiales are usually the most abundant in aerobic biomass. However, Pseudomonales have the capacity of growing in limited media. In other words, this phylum which showed greater proportion at genus level (56.10%) could suppress the development of taxa as *Clostridium* including bacteria in charge of ammonium oxidation in nitrifying conditions as *Nitrosomonas* spp.

## Conclusion

In this paper, a fixed-film systems combining both biofilm and suspended biomass were considered to evaluate SND process. Polyethylene rings was selected as the best support with removal organic matter and nitrogen efficiencies greater than 70 and 95%, respectively. Regardless of the support material, maximum specific nitrifying activity in the suspended biomass was 88% higher than the activity in fixed biomass. Maximum specific denitrifying activities were higher in the polyethylene rings (5.3 ± 0.34 mg of NO_3_-N/g TVS∙h) than polyurethane foam (4.64 ± 0.13 mg of NO_3_-N/g TVS∙h), associated to the depth of the developed biofilm. The SND processes was achieve through the following reasons: approximately 30 ± 1% of nitrogen compounds were transformed to molecular nitrogen, supported by the low C/N ratio of the treated influent, and according with the molecular analysis, 50% of bacterial genus are associated to *Pseudomonas* contributing to both heterotrophic nitrification and aerobic denitrification.

## Abbreviations

ANAMMOX: Anaerobic ammonium oxidation
ANOVA: Analysis of variance
AOB: Ammonia oxidizing bacteria
ASV: Amplicon sequence variant
COD: Chemical oxygen demand
DMSO: Dimethylsulfoxide
DO: Dissolved oxygen
FB: Fixed biomass
HRT: Hydraulic retention time
MBBR-MBR: Mobile packed-bed bioreactors coupled to membrane bioreactors
MSDA: Maximum specific denitrification
MSNA: Maximum specific nitrification
NH_4_^**+**^: Ionised ammonia
NH_3_^**+**^: Unionised ammonia
NO_2_^**−**^: Nitrite
NO_3_^−^: Nitrate
NOB: Nitrate by oxidizing bacteria
NR: No reported
OLR: Organic load rate
PCR: Polymerase chain reactions
PE: Polyethylene
dNTP: Deoxynucleoside triphosphate
PUR: Polyurethane
PVC: Polyvinylchloride
R_1_: Polyurethane foam
R_2_: Polyethylene rings
SB: Suspended biomass
SD: Standard deviation
SND: Simultaneous nitrification–denitrification
SHARON: Single reactor system for high ammonium removal over nitrite
TS: Total solids
TVS: Total Volatile Solids
UASB: Upflow Anaerobic Sludge Blanket
VSS: Volatile suspended solids
VRR: Volumetric removal rate
