# Author Correction: Organic matter removal in a simultaneous nitrification–denitrification process using fixed-film system

**DOI:** 10.1038/s41598-022-23965-5

**Published:** 2022-11-17

**Authors:** P. González-Tineo, A. Aguilar, A. Reynoso, U. Durán, M. Garzón-Zúñiga, E. Meza-Escalante, L. Álvarez, D. Serrano

**Affiliations:** 1 Departamento de Ciencias del Agua y Medio Ambiente, Instituto Tecnológico de Sonora, Calle 5 de febrero 818 Sur. Col. Centro., Cd. Obregón, Sonora Mexico; 2 Instituto de Ingeniería, UNAM, P.O. Box 70-186, 04510 México City, Mexico; 3 Instituto Politécnico Nacional (IPN) CIIDIR-DURANGO, Sigma 119, 20 de Noviembre II, 34220 Durango, Mexico; 4 Departamento de Ciencias Agronómicas y Veterinarias, Instituto Tecnológico de Sonora, Cuidad Obregón, Mexico

Correction to: *Scientific Reports* https://doi.org/10.1038/s41598-022-05521-3, published online 03 February 2022

The original version of this Article contained an error in Affiliation 3, which was incorrectly given as:

Centro Interdisciplinario de Investigación para el Desarrollo Integral Regional (CIIDIR), Sigma 119, 20 de noviembre II, 34220 Durango, Mexico.

The correct Affiliation is listed below:

Instituto Politécnico Nacional (IPN) CIIDIR-DURANGO, Sigma 119, 20 de noviembre II, 34220 Durango, Mexico.

The original Article has been corrected.

